# Molecular Mechanisms of Mucormycosis Pathogenesis: Host–Pathogen Interactions and Immune Evasion

**DOI:** 10.3390/pathogens15050522

**Published:** 2026-05-12

**Authors:** Awadh Alanazi, Mohamed N. Ibrahim, Maram Awied Alenezi, Wejdan Oudah Albalawi

**Affiliations:** 1 Department of Clinical Laboratory Sciences, College of Applied Medical Sciences, Jouf University, Sakaka 72388, Saudi Arabia; 2 Department of Clinical Laboratories Sciences, College of Applied Medical Sciences at Al Qurayyat, Jouf University, Al Qurayyat 77454, Saudi Arabia; mnabil@ju.edu.sa (M.N.I.);; 3 Northern Borders Health Cluster, Almosaediah, Arar 73311, Saudi Arabia

**Keywords:** mucormycosis, host–pathogen interactions, immune evasion, CotH–GRP78 axis, antifungal targets, host immune response

## Abstract

Mucormycosis, triggered by fungi of the order Mucorales, represents a potentially fatal invasive mycosis, with death rates over 50% despite intensive therapy. The COVID-19 pandemic brought a sharp increase in cases, especially in individuals with diabetes mellitus and those undergoing immunosuppressive treatment, emphasizing significant gaps in our comprehension of disease pathogenesis. Emerging molecular studies have highlighted key virulence factors, such as the CotH family of invasins that facilitate endothelial invasion via interaction with glucose-regulated protein 78 (GRP78), complex iron acquisition systems necessary for fungal growth, and the release of mucoricin, a ricin-like toxin that impairs vascular integrity. Host defense depends mainly on innate immunity, with neutrophils and macrophages working as critical effector cells, while adaptive Th1 and Th17 responses aid in the fungal removal. Mucorales use a variety of immune evasion techniques, such as pathogen-associated molecular pattern (PAMP) masking via cell wall transformations, resistance to phagocytic death, and metabolic utilization of host factors including hyperglycemia and increased free iron in diabetic ketoacidosis (DKA). This review summarizes current evidence of the molecular processes underlying mucormycosis pathogenesis, underscoring host–pathogen interactions at the cellular and molecular levels, immune evasion tactics, and translational potential for new diagnostic and therapeutic approaches. Comprehending these molecular processes is crucial for creating efficient therapies against mucormycosis in an era of growing immunocompromised patients and expanding infectious disease synergies.

## 1. Introduction

Mucormycosis is an uncommon but extremely deadly invasive fungal infection that is brought on by filamentous fungi of the Mucorales order, which include more than 40 pathogenic species, primarily belonging to genera such as *Rhizopus*, *Mucor*, and *Lichtheimia* species. Despite being thought of as rare, its prevalence has grown globally over the last 20 years, especially in immunocompromised individuals [1,2]. While population-based projections from North America and Europe indicate an annual incidence of 0.01 to 0.2 cases per 100,000 people, significantly higher instances (approximately 70 times) have been documented in India, which is primarily due to the high rates of untreated diabetes mellitus [1]. Amidst the COVID-19 pandemic, India documented thousands of cases of mucormycosis linked to the virus, underscoring the close relationship among immune dysregulation, corticosteroid exposure, and hyperglycemia [1]. The total death rates range from 40% to exceeding 50%, over 80% in disseminated illness [1,2]. The clinical manifestations of mucormycosis include extensive tissue necrosis, vascular thrombosis, and angioinvasion. Rhino-orbito-cerebral and pulmonary forms are the most common, and the site of infection and underlying host factors have a significant impact on the outcome. Untreated diabetes mellitus, especially diabetic ketoacidosis (DKA), hematologic cancers, solid organ and hematopoietic stem cell transplantation, an excess of iron, extended neutropenia, and long-term or high-dose corticosteroid treatment are key risk factors [3]. Recent years have seen a significant improvement in our knowledge of the molecular pathogenesis of mucormycosis thanks to the use of advanced in vitro and in vivo model systems, genomic sequencing, and genetic manipulation technologies like CRISPR-Cas9 [3]. These studies have shown that a complex interaction between host susceptibility elements and fungal virulence factors determines Mucorales pathogenicity [3,4]. At the molecular level, important virulence processes include tailored invasion proteins, complex iron acquisition systems, released hydrolytic enzymes, and adaptive reactions to host environmental stresses. Simultaneously, host defenses include coordinated innate and adaptive immune reactions that, when weakened, allow quick fungal growth and tissue destruction [4].

A number of crucial determinants are at the center of the molecular architecture of Mucorales virulence. The CotH (spore coat protein homolog) family of cell surface proteins facilitates fungal attachment to and penetration of endothelial cells via particular interactions with the host receptor glucose-regulated protein 78 (GRP78), also known as binding immunoglobulin protein (BiP), a stress-inducible endoplasmic reticulum chaperone [4]. This pathway is significantly elevated in hyperglycemic and ketoacidotic conditions. Iron acquisition is another essential virulence mechanism, with Mucorales having high-affinity iron uptake systems, such as the FTR1 permease and siderophore-driven processes, that utilize the increased free iron availability feature of DKA and serious COVID-19 [5]. Furthermore, Mucorales release an arsenal of proteolytic, lipolytic, and glycosidic enzymes that promotes tissue penetration and vascular invasion [5,6]. Numerous cellular and molecular elements are involved in the host immune response to Mucorales. The main innate immune defenses are neutrophils and macrophages, which use phagocytosis, oxidative burst mechanisms, and neutrophil extracellular trap (NETs) formation to get rid of fungal spores and hyphae [6]. Fungal detection and inflammatory signaling processes are mediated by pattern recognition receptors (PRRs), such as C-type lectin receptors (CLRs) and Toll-like receptors (TLRs). The protective immunity towards mucormycosis is aided by adaptive immunity, specifically the Th1 and Th17 responses that produce interleukin-17 (IL-17) and interferon-gamma (IFN-γ) [6,7]. However, Mucorales have developed complex immune escape methods which undermine these host defenses. These tactics include cell wall remodeling to decrease exposure to pathogen-associated molecular patterns (PAMPs), metabolic adaptations that allow proliferation in hostile host microenvironments, durability against oxidative damage and modification of phagosome maturation [7].

This narrative review summarizes what is known at present about the molecular processes underlying the pathogenesis of mucormycosis. The biological and molecular underpinnings of virulence are examined, host–pathogen interactions are broken down at the cellular and molecular levels, host immune responses and fungal immune evasion tactics are reviewed, and translational implications for diagnosis and treatment are discussed. It seeks to offer a thorough framework for comprehending this debilitating infection and to pinpoint fruitful therapeutic intervention approaches.

## 2. Biological and Molecular Basis of Virulence

Mucorales are early-diverging filamentous fungi that belong to the subphylum *Mucoromycotina*. They include therapeutically important species like *Rhizopus*, *Mucor*, *Lichtheimia*, *Rhizomucor*, and *Cunninghamella*; with *Rhizopus arrhizus* causing most invasive infections globally [8]. Mucorales pathogenicity results from a coordinated interaction between genomic architecture, structural modifications, and specialized virulence factors, which together allow for fast tissue invasion and angioinvasive disease progression. Comparative genomics has shown somewhat big genomes (≈35–50 Mb) with high repeat density and proof of ancestral whole-genome duplication, especially in *Rhizopus arrhizus* var. *delemar* [8]. This genomic enlargement is linked with enrichment of gene families involved in iron acquisition (e.g., permease), siderophore transport, released aspartyl proteases, lipases, and carbohydrate-active enzymes that promote tissue penetration and metabolic adaptation under host-induced nutrient restriction. Notably, the CotH gene family is distinctly enlarged in Mucorales and directly facilitates endothelial invasion via interaction with host GRP78, acting as a molecular determinant of angioinvasion [4]. These organisms’ ability to adapt is further strengthened by the existence of RNA interference-dependent epimutational processes, which also lead to phenotypic plasticity and transient antifungal resistance.

The virulence of some Rhizopus strains may also be influenced by endosymbiotic bacteria, albeit the evidence to date seems to be strain dependant. Earlier investigations found no discernible role of bacterial endosymbionts in mucormycosis models while more recent research showed that endosymbionts in a clinical *R. microsporus* isolate increased fungal virulence and phagocyte evasion [9].

Crucially, in this study, the term “pathogenic Mucorales” refers to Mucorales species that might cause invasive disease in vulnerable hosts, especially in situations like diabetes mellitus, ketoacidosis, neutropenia, exposure to corticosteroids, or other immune dysfunction. Thus, rather than being the result of a single exclusive fungal characteristic, pathogenicity should be regarded as a multifactorial and host-dependent process [4].

Non-pathogenic or environmental Mucorales species share many of the characteristics covered in this review, such as chitosan-rich cell walls, quick spore germination, and adaptation to aerobic or hypoxic conditions. As a result, these traits by themselves shouldn’t be considered conclusive pathogenic determinants. Rather, the coordinated expression of several virulence-associated mechanisms, such as endothelial adhesion and invasion, effective iron acquisition, stress adaptation, immune evasion, and angioinvasive growth within susceptible hosts, appears to be necessary for invasive pathogenicity in clinically relevant Mucorales [4,8]. Together, these biological characteristics aid in adaptability and survival in the host environment.

Mucorales start infection via inhaled or traumatically introduced sporangiospores that should quickly adjust to the host environment to spread disease. A crucial factor in this process is the fungal cell wall, which is rich in chitin and especially chitosan [10]. High chitosan content prevents immunogenic β-glucans from being exposed to the surface, which reduces their detection by host PRRs such Dectin-1 and attenuates early innate immune activation while preserving structural integrity during hyphal expansion [10]. Dynamic cell wall modifications additionally allow masking of PAMPs (β-1,3-glucans and chitin) while responding to host immune pressure. Melanin-like pigments that are deposited in hyphae and spores improve persistence in inflammatory tissues by offering extra defense against phagocyte-induced injury and oxidative damage. Thermotolerance is also crucial for virulence, facilitated by stress-response processes such as heat shock proteins and calcineurin-dependent signaling, which support growth at physiological temperature [11]. After adjusting to host circumstances, Mucorales use specialized host–fungal interactions to start tissue invasion.

These structural and adaptive characteristics allow subsequent host invasion via specific molecular interactions. The CotH protein family works as a crucial virulence factor, acting as fungal invasins. While non-pathogenic or less pathogenic Mucorales appear to have fewer or less enlarged CotH family members, highly virulent species, such as *Rhizopus arrhizus* var. *delemar*, express multiple CotH paralogs [12]. CotH3 facilitates attachment to host GRP78 on nasal epithelial and endothelial cells, promoting receptor-dependent endocytosis. CotH7, in comparison, engage with integrin β1 on alveolar epithelial cells and stimulates EGFR signaling, facilitating pulmonary invasion [13]. Host metabolic factors, such as hyperglycemia, acidosis, and enhanced free iron, elevate both fungal CotH expression and host receptor availability, thereby enhancing the invasion efficiency [3]. After adhesion and penetration, Mucorales use a variety of released hydrolytic enzymes to enter tissue barriers. Aspartyl proteases and metalloproteases break extracellular matrix proteins and basement membranes, allowing hyphal extension across tissue planes. Lipases additionally take part by degrading host lipid membranes and supporting fungal metabolic needs during invasive proliferation. Studies using transcriptomics that show these enzymes are induced during infection lend credence to their involvement in the active course of disease. Furthermore, the release of mucoricin, a toxin that resembles ricin, directly damages endothelium by preventing host protein synthesis, which leads to tissue necrosis and increased vascular permeability [14]. Together, these virulence-related processes allow Mucorales to coordinate host survival, metabolic adaptability, and tissue invasion. Iron acquisition systems enhance long-term fungal growth, hydrolytic enzymes aid in tissue penetration, and stress-response signaling pathways encourage persistence under unfavorable host microenvironmental settings.

## 3. Host–Pathogen Interactions: From Entry to Tissue Invasion

### 3.1. Spore Adhesion and Endothelial Invasion

The transformation of Mucorales from environmental saprophytes to invasive pathogens starts with spore deposition on respiratory or sinonasal mucosa, after which they adhere, germinate, and penetrate host barriers. Receptor–ligand interactions, such as the CotH–GRP78 axis discussed in Section 2, mediate these early processes. GRP78, a stress-inducible endoplasmic reticulum chaperone, moves to the cell surface under nmetabolic and oxidative damage, where it act as an invasion receptor. Receptor-mediated endocytosis and cytoskeletal rearrangements that allow fungal internalization into endothelial cells are facilitated by interacting with fungal CotH proteins [15,16]. GRP78 is a crucial host determinant of endothelial entrance, as evidenced by the constant correlation between its surface expression on endothelial cells and heightened vulnerability to fungal invasion [3]. Particularly, Mucorales’ receptor activation is context-dependent and different host receptors contribute to invasion in a tissue-specific way, indicating functional diversification within the CotH protein family. Section 2 describes the mechanistic components of integrin β1–EGFR signaling in pulmonary epithelial invasion. Together, these outcomes lend credence to the idea that Mucorales use different host entrance pathways based on the cellular milieu. These initial invasion episodes eventually lead to vascular involvement and tissue dispersion. After internalization, angioinvasion, enhanced vascular permeability, and endothelial damage promote hematogenous dispersion [17]. Receptor-mediated entrance is therefore a precursor to invasive illness.

### 3.2. Iron Acquisition as a Central Pathogenic Axis

**Figure 1:**
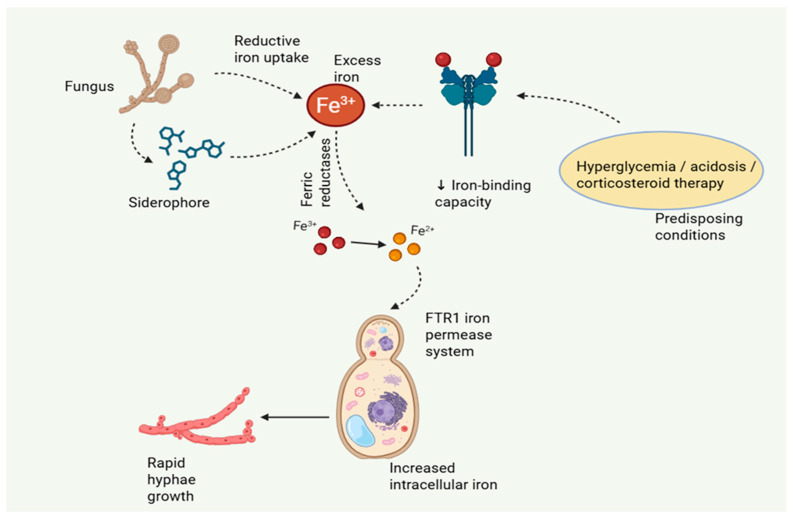
Mucorales iron acquisition strategies.

Iron homeostasis is central to the risk to mucormycosis. The host maintains “nutritional immunity” under physiological conditions by tightly enclosing iron in lactoferrin, ferritin, transferrin, and heme, keeping free iron concentrations at levels that are incompatible with the growth of microorganisms. Rarely do mucorales induce illness in this iron-deficient environment. However, clinical conditions that interfere with iron regulation produce a metabolic milieu that is particularly conducive to fungal growth [18]. DKA is the most obvious example. The iron-binding ability of ferritin and transferrin is reduced by acidotic pH, which raises the amount of free iron in the blood. Concurrently hyperglycemia and increased ketone bodies supply extra metabolic substrates, while acidosis increases fungal expression of virulence factors. This combination of iron overload and metabolic dysfunction clarifies why DKA and mucormycosis are so closely related [11]. Similar to this, deferoxamine treatment unexpectedly increases the risk of infection because ferroxamine, a component of the deferoxamine–iron complex, acts as a xenosiderophore that Mucorales can easily employ to supply iron to the pathogen. Mucorales uses high-affinity iron acquisition mechanisms at the molecular level to take advantage of iron-replete environments. The FTR1 iron permease, in conjunction with surface ferric reductases, facilitates effective uptake of reduced iron and is important for full virulence in experimental models [5,19]. Furthermore, these fungi can utilize exogenous siderophores, highlighting their metabolic adjustments (Figure 1).

The importance of iron in the development of disease has been highlighted by experimental investigations that show a clear correlation between increased iron availability and increased fungal growth and pathogenicity in vivo. Simultaneously, high iron levels have a negative impact on host immunological function by reducing neutrophil oxidative processes and macrophage-mediated antifungal activity [20]. Iron availability is a key pathogenic element in mucormycosis because it both promotes fungal growth and weakens host defense mechanisms. As a result, iron serves as both a source of nutrients and a crucial factor in determining the severity of disease, connecting host metabolic dysregulation to weakened immune function and heightened fungal invasiveness.

### 3.3. Angioinvasion and Thrombosis

Angioinvasion is the decisive factor that turns localized mucormycosis into a quickly worsening, potentially fatal condition. Mucorales hyphae show a strong affinity for vascular structures and penetrate deeper tissues following initial epithelial invasion. Section 3.1 describes the receptor-mediated pathways that begin endothelial penetration. Hyphal extension over the vascular barrier causes direct damage to vessel walls and promotes vascular invasion after endothelial contact. Intravascular occurrence of hyphae triggers a strong procoagulant reaction [21,22]. Endothelial damage facilitates tissue factor expression and stimulation of the extrinsic coagulation cascade, leading to fibrin deposition and thrombus development. Direct contact between fungal elements and platelets additionally elevate aggregation and luminal occlusion. Progressive thrombosis impairs blood flow, resulting in tissue ischemia and infarction, clinically represented by the distinctive black necrotic lesions. Crucially, in addition to destroying tissue, vascular occlusion hinders the movement of immune cells and the delivery of antifungal medications. Since this ischemic niche permits continuous fungal survival and growth, angioinvasion and thrombosis are identified as key, self-amplifying factors that influence the extent of mucormycosis [23] (Table 1).

## 4. Host Immune Responses to Mucorales

### 4.1. Innate Immunity

The basis of host defense against Mucorales is innate immunity, which plays a major role in determining whether inhaled or inoculated spores are eradicated or develop into an invasive disease. This idea is strongly supported by clinical epidemiology, which emphasizes the importance of intact innate responses by showing that the most probable risk factors for mucormycosis are corticosteroid exposure, uncontrolled diabetes mellitus, neutropenia, and qualitative neutrophil defects. The predominant effector cells in antifungal response are neutrophils. By using opsonic receptors and PRRs, they are able to identify Mucorales and quickly phagocytose spores and tiny hyphal components [35]. The primary mechanism for intracellular killing is the activation of the nicotinamide adenine dinucleotide phosphate (NADPH oxidase) complex, which produces superoxide anions and downstream ROS, such as hypochlorous acid and hydrogen peroxide. The proteins, nucleic acids, and fungal membranes are fatally damaged by these oxidants. People with chronic granulomatous disease are particularly vulnerable because they do not have functional NADPH oxidase, which highlights the vital role of oxidative processes. Neutrophils also use non-oxidative processes like degranulation, which releases proteolytic enzymes, defensins, and cathelicidins that compromise the integrity of fungal cells [36]. In response to hyphae that are too big for phagocytosis, NETs which are web-like structures made of DNA and antimicrobial proteins that immobilize fungal filaments and target fungicidal effectors. NETs aid in hyphal suppression, but too much of them can cause tissue damage and thrombosis, underscoring the fine line between immunopathology and protection [6].

An equally important first line of defense is offered by macrophages, especially in cases of pulmonary mucormycosis. The earliest layer of defense for inhaled spores is provided by alveolar macrophages, which engulf them and prevent germination in acidified phagolysosomes that are rich in hydrolytic enzymes and reactive oxygen and nitrogen intermediates. Achieving effective early clearance at this point is essential to halting the development of invasive, angioinvasive hyphal growth. Polarization state and macrophage function are closely related [37]. Pro-inflammatory cytokines, such as TNF-α, IL-1β, and IL-12, are secreted by classically activated (M1) macrophages, which improve fungicidal activity and promote antifungal immunity. Alternately activated (M2) macrophages, on the other, prefer tissue repair and exhibit a decreased capacity for microbicidal activity [18].

PRRs, such as CLRs like Dectin-1 and Dectin-2 and TLRs (TLR2 and TLR4), are responsible for coordinating fungal detection. Crucially, while respiratory epithelial cells play a role in early pathogen detection, professional innate immune cells, such as neutrophils, dendritic cells, and macrophages, are the main mediators of fungal recognition and subsequent inflammatory reactions. They identify conserved cell wall carbohydrates and trigger nuclear factor kappa B (NF-κB) dependent inflammatory signaling. Complement activation through lectin and alternative pathways produces C3a and C5a, which recruit and activate more immune cells and improve opsonization [17].

**Figure 2:**
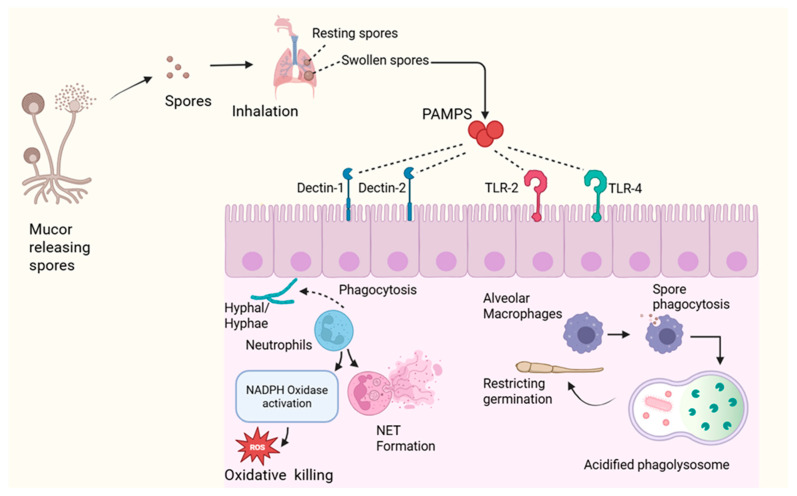
Host Innate Immune Response to Mucorales Infection.

Crucially, new research shows that innate immunity in mucormycosis is severely dysregulated rather than just suppressed, especially in high-risk conditions like COVID-19. A retrospective case–control study in COVID-19-associated mucormycosis (CAM) showed a significant increase in pro-inflammatory cytokines (IFN-γ, IL-1β, IL-18, MCP-1) and a decrease in cytotoxic CD56^+^CD16^+^ NK cells, suggesting a paradoxical state of increased inflammation and compromised cytotoxic effector function [38]. This imbalance implies that while inflammatory signaling is enhanced, important antiviral and antifungal cytotoxic mechanisms are weakened. Multi-omics investigations in COVID-19-associated pulmonary mucormycosis (CAPM) frequently demonstrate upregulation of fungal recognition pathways, like Dectin-2, macrophage C-type lectin (MCL), FcRγ-associated receptors, and CLEC-2, as well as increased expression of complement receptors, enhanced NETosis, and heightened inflammatory mediators, like S100A8/A9, lipocalin, and MMP9 [39]. Ironically, mTOR and MAP kinase pathways were abnormally elevated in monocytes, while important downstream signaling pathways necessary for efficient antifungal defense, such as JAK–STAT, CARD9, and IL-17 signaling, were simultaneously downregulated. This pattern points to a hyperinflammatory but functionally compromised state of innate immunity. Accordingly, transcriptome profiling of host innate immune cells and infected lung tissues in pulmonary mucormycosis has revealed the activation of genes related to iron metabolism, PRR signaling, cytokines, and chemokines, suggesting the involvement of both innate and adaptive immunological mechanisms [39,40]. Proteomic data, however, show that these signals are only partially translated into functional immune responses, with host defense proteins and pathways linked to cytoskeletal architecture and cell junction integrity being globally suppressed [40]. Despite apparent immunological stimulation, this discrepancy between transcriptional activation and protein-level execution reveals an ineffective immune defense. Together, these results imply that, despite increased inflammatory signals, innate immunity in mucormycosis is not only reduced but also dysregulated in severe or predisposed stages, leading to inefficient fungus clearance (Table 2) (Figure 2).

### 4.2. Adaptive Immunity

Although innate immunity plays a pivotal role in the initial containment of Mucorales, adaptive immune responses offer crucial immunological coordination and have the potential to impact the course and outcome of the disease. Among adaptive elements, CD4 T helper (Th) cell subsets, especially Th1 and Th17, have become the main agents of cellular immunity against fungal infections. Th1 responses are distinguished by the release of IFN-γ, a cytokine essential for phagocyte-driven killing enhancement and macrophage stimulation. IFN-γ increases oxidative burst dependent on NADPH oxidase, enhances classical (M1) macrophage polarization, and upregulates antimicrobial pathways, such as inducible nitric oxide synthase [35]. By these means, Th1 immunity reinforces innate confinement of spores and hyphae and increases the fungicidal potential of neutrophils and macrophages. Experimental approaches of pulmonary mucormycosis show enhanced fungal clearance when IFN-γ signaling is intact, indicating that Th1-driven responses play a protective role [43]. Additionally, IFN-γ maintains integrated antifungal defense by amplifying local inflammatory circuits and facilitating efficient communication between antigen-presenting cells and effector leukocytes.

Additional protection is offered by Th17 responses, especially at the interfaces between mucosa and epithelium. At fungal invasion sites, Th17 cells generate the cytokines IL-17A, IL-17F, and IL-22, which improve neutrophil recruitment, activation, and survival. IL-17 promotes the release of chemokines and antimicrobial peptides by stromal and epithelial cells, whereas IL-22 supports the integrity and repair of the epithelial barrier. Th17 differentiation is supported by IL-23 derived from dendritic cells, which connects adaptive polarization and innate fungal recognition [44]. However, mounting data suggests that mucormycosis impairs this beneficial Th17 axis. Despite increased upstream inflammatory responses, transcriptome studies show downregulation of IL-17 signaling pathways, indicating a functional deficiency rather than merely immune suppression [39]. Accordingly, a case–control study found that patients with CAPM had much lower levels of circulating Th17 cells, which further supports compromised Th17-mediated antifungal immunity [45]. Patients with diabetic rhino-orbital-cerebral mucormycosis in another observational case-based investigation showed a significant immunological imbalance with decreased regulatory T cells and increased Th17 cells [46].

While the exact extent of Th17 involvement in mucormycosis is still being studied, more general antifungal data suggest that IL-17-associated processes are crucial for maintaining neutrophil-dominated immunity, particularly in infections of the mucosa and airways. The host immune profile, fungal load, and anatomical site all probably affect the relative contributions of Th1 and Th17 reactions [47].

Although CD8 T cells may play a part in mucormycosis via cytokine release and cytotoxic activities, their exact function is unclear. Clinical findings indicate that isolated lymphopenia carries a lesser risk than neutropenia, which is significant because it highlights the fact that adaptive immunity, while helpful, cannot replace intact innate defenses [48].

Qualitative deficiencies in T-cell immunity are further highlighted by recent clinical data. Regardless of COVID-19 status, a comparative case–control research showed that patients with mucormycosis had both CD4 and CD8 T cell depletion. Additionally, T-cell fatigue was more severe in non-COVID individuals, as evidenced by increased expression of the inhibitory receptor LAG-3 on CD4^+^ and CD8^+^ T cells and substantial co-expression of exhaustion markers PD-1/LAG-3 and LAG-3/TIM-3 on CD8^+^ T cells. These results suggest that functional exhaustion of T cells may significantly decrease adaptive antifungal immunity in addition to numerical depletion [47]. Simultaneously, another retrospective case–control study showed decreased degranulation capacity and impaired T-cell cytotoxicity, as well as an inverse relationship between increased systemic cytokines (IFN-γ and IL-18) and CD4^+^ T-cell activity. According to this research, antifungal immunity is further compromised by a paradoxical condition where systemic hyperinflammation coexists with functionally depleted and inefficient T-cell responses [38].

**Figure 3:**
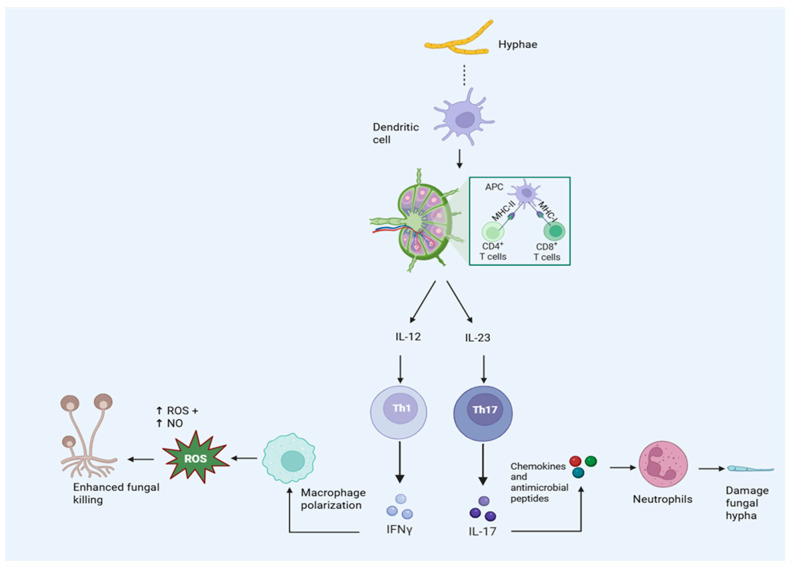
Host adaptive Immune Response to Mucorales Infection.

Despite humoral reactions to mucorales arise during infection, it is unclear if they are protective in natural disease. Nonetheless, animal studies showing protection with monoclonal antibodies against CotH proteins suggest that antibodies can improve complement-mediated clearance, opsonophagocytosis, and fungal invasion [16]. Together, adaptive immunity strengthens and maintains antifungal responses, influencing the removal of pathogens and the course of inflammation (Figure 3).

## 5. Immune Evasion Strategies

### 5.1. Masking and Modulation of PAMP Recognition

A key strategy used by Mucorales to circumvent host immunity is the deliberate modification of their cell wall to reduce PRRs recognition. Since innate immune cells first come into contact with the fungal cell wall, the composition of this wall directly influences the intensity and caliber of host inflammatory reactions. This interface is specifically designed in Mucorales to limit contact with PAMPs that are highly immunostimulatory [7]. A crucial structural element of the Mucorales cell wall, chitosan supports both fungal integrity and host contact. Its existence in the cell wall has been linked to persistence in host tissues and resistance to host defenses.

Chitosan can conceal more immunogenic components of the cell wall and is less effectively recognized by host PRRs. More specifically, there is relatively little surface exposure of β-1,3-glucans, which are strong ligands for the C-type lectin receptor Dectin-1 [49]. Since Dectin-1 involvement is a key process driving NF-κB stimulation, pro-inflammatory cytokine generation, and phagocyte stimulation, decreased β-glucan availability results in reduced innate immune signaling [9]. This structural feature not only promotes immune escape during early infection but also explains the low sensitivity of serum β-D-glucan tests in mucormycosis compared to other invasive fungal illnesses. This line of defense is further strengthened by melanization. Antioxidant-capable melanin-like pigments found in spore and hyphal walls neutralize ROS produced during neutrophil and macrophage oxidative burst. Melanization has also been linked to lowered intracellular killing capacity and impaired phagosome maturation, which increases fungal survival in host tissues.

Crucially, the composition of the cell wall in Mucorales is dynamically controlled in reaction to immunological pressure and environmental factors. This remodeling makes it possible to modify PAMP exposure in a context-dependent manner at various infection stages [33]. Melanization, limited β-glucan display, and chitosan enrichment work together as a cohesive strategy to impede immune detection without compromising structural integrity or invasive capacity.

### 5.2. Resistance to Phagocytic Killing

Although neutrophil and macrophage phagocytosis is a key antifungal defense, Mucorales have developed a number of defense mechanisms that allow survival even in the face of effective uptake. The hostile phagolysosomal environment, which is typified by acidification, hydrolytic enzymes, and an increase in reactive oxygen and nitrogen species produced during oxidative burst, is presented to fungal spores after internalization. Mucorales use an integrated antioxidant defense system to cope with this stress [50,51]. Superoxide dismutases produce hydrogen peroxide from superoxide anions, which is then eliminated by catalases and peroxidases. Concurrently, systems that rely on glutathione and thioredoxin preserve intracellular redox equilibrium and aid in the restoration of oxidatively damaged proteins and lipids. Transcriptional analysis showed induction of oxidative damage response genes during phagocytosis, and degradation of important antioxidant enzymes decreases virulence in experimental models, highlighting their pathogenic significance [52].

An extra layer of defense is offered by pigments that resemble melanin and are integrated into the cell wall. These polymers have the ability to scavenge free radicals, which can lessen the effectiveness of intracellular killing and mitigate the effects of oxidants derived from phagocytes. Even though the exact mechanisms are still unclear. Spores have been seen to survive inside macrophages in specific circumstances, indicating partial tolerance of phagolysosomal stress rather than total eradication. The interaction changes from intracellular inhibition to extracellular battle during this phase, which frequently leads to collateral tissue damage and unsuccessful phagocytosis [53]. In addition to their biochemical resistance, Mucorales use a structural escape mechanism. Despite the fact rapid germination and filamentous growth are typical biological traits of Mucorales and are not limited to pathogenic species, the change to elongated hyphal forms during infection makes it more difficult for phagocytes to efficiently engulf and destroy fungal cells [50]. Collectively, oxidative damage resistance, partial intracellular survival, and swift morphologic transition allow Mucorales to evade phagocyte-driven clearance during the crucial early stages of infection.

### 5.3. Metabolic Adaptation in Host Microenvironments

One known risk factor for mucormycosis is metabolic dysregulation in the host, especially when DKA is present. Hyperglycemia impairs innate immune defenses and increases mucorales growth, according to both clinical observations and experimental research. Early antifungal defenses are diminished by increased glucose concentrations due to impaired neutrophil activity. High glucose simultaneously encourages fungal growth and increases sensitivity to tissue invasion, according to evidence from both in vitro and in vivo investigations [54,55]. Acidosis also raises the likelihood of infection by increasing iron availability, which encouraged Mucorales development and pathogenicity [18,56]. Moreover, increased fungal growth is linked to ketone molecules like β-hydroxybutyrate, which build up during DKA [57]. A characteristic of mucormycosis, angioinvasion causes localized hypoxia, tissue necrosis, and vascular thrombosis. Research shows that Mucorales can continue to grow when oxygen tension is lower, which helps them persist in ischemic tissues [58]. All of these results point to metabolic adaptation to acidic, hypoxic, iron-replete, and hyperglycemic microenvironments as a key element in the pathophysiology of mucormycosis.

### 5.4. Modulation of Host Signaling Pathways

Mucorales deliberately alter intracellular signaling pathways within the host in order to initiate infection and thwart immune responses. Interactions between pathogen and host receptors not only make entry easier but also start signaling cascades that alter inflammatory and survival processes. According to experimental data, Mucorales modify NF-κB signaling, a key regulator of proinflammatory gene transcription, which may reduce the effectiveness of cytokine responses and hinder coordinated innate immunity This alteration is biologically associated with decreased TLR2/TLR4–MyD88 signaling and decreased Dectin-1–SYK activation owing to β-glucan masking, which together result in decreased nuclear translocation of NF-κB and impaired generation of TNF-α and IL-6 [36]. Simultaneously, the PI3K/Akt pathway’s activation or dysregulation affects endothelial barrier function, metabolic adaptation, and cell survival, all of which promote fungal survival. Recent data also indicates that fungal effectors that are secreted may have a direct impact on the polarization of immune signaling. Induction of STAT6 phosphorylation, for example, has been linked to the skewing of macrophages toward an M2 phenotype, which is distinguished by tissue-remodeling and anti-inflammatory properties as opposed to fungicidal activity [59]. By means of coordinated disruption of inflammatory, survival, and polarization processes, Mucorales modify host cellular reactions at the signaling level. This type of modulation is a crucial immune evasion tactic and plays a significant role in the development and intensity of mucormycosis.

## 6. Translational Implications

### 6.1. Molecular Diagnostics

Although early detection of mucormycosis is crucial for increasing survival, clinical practice continues to face this obstacle. Clinically and radiologically, the illness frequently manifests as vague symptoms that are similar to those of other invasive mold infections. Traditional diagnostic methods, such as direct microscopy, histopathology, and culture, are often delayed in providing a conclusive diagnosis and prompt antifungal treatment due to their variable sensitivity and slow turnaround times. As a result, molecular diagnostics have become crucial auxiliary tools. Fungal DNA can be detected more quickly and sensitively from tissue biopsies and bronchoalveolar lavage fluid thanks to polymerase chain reaction (PCR) based assays that concentrate on genomic regions particular to Mucorales, especially 18S rRNA and internal transcribed spacer (ITS) sequences [60,61]. Additionally, circulating fungal DNA has occasionally been found in serum, indicating the possibility of less intrusive testing. Even though standardized procedures and validated diagnostic thresholds have not yet been established, real-time quantitative PCR may facilitate treatment monitoring and offer an estimate of fungal burden.

The therapeutic usefulness of these methods has been greatly enhanced by recent clinical research. For example, serum Mucorales qPCR demonstrated good diagnostic accuracy (sensitivity 85.2%, specificity 89.8%) in the MODIMUCOR prospective study and allowed for earlier diagnosis by a median of 4 days compared to traditional mycological or imaging approaches. An 85% decrease in 30-day mortality was linked to early qPCR negative following the start of treatment [62]. According to a systematic review and meta-analysis, PCR is a good diagnostic tool for mucormycosis, albeit sample type affects how sensitive it is. It is greatest in bronchoalveolar lavage fluid, followed by blood (approximately 81%) and tissue (about 86%), while specificity is constantly high (around 90–96%) for all specimen types [63]. The MucorGenius^®^ PCR assay showed excellent sensitivity for mucormycosis detection in a multicenter cohort study, reaching 94.4% in probable and confirmed cases. In addition to identifying Mucorales DNA in biopsy and pulmonary samples, it also detected additional instances and coinfections that were overlooked by standard diagnostic techniques [64]. Clinical contexts are turning to quantitative PCR-based detection of circulating Mucorales DNA, which may help with fungal burden estimation and treatment response monitoring. Despite continuous efforts to increase consistency and clinical utility, variations in specimen type, DNA extraction techniques, test targets, and positivity thresholds still have an impact on inter-laboratory reproducibility and wider clinical standardization [63,65].

Metagenomic next-generation sequencing (mNGS) is becoming a potent, objective diagnostic tool that can identify a variety of diseases directly from clinical specimens, going beyond targeted PCR techniques. When compared to conventional fungal detection techniques, mNGS technology is quicker, more sensitive and specific, and capable of precisely identifying species, even subspecies, as well as uncommon fungi and other infections. Growing clinical evidence demonstrates its translational significance in mucormycosis. Early diagnosis made possible by mNGS was linked to better survival in a multicenter retrospective investigation of ICU patients, whereas delayed diagnosis was found to be an independent predictor of mortality [66]. In a different single-center investigation of liver transplant recipients, mNGS consistently yielded the earliest diagnosis in every case of mucormycosis, ahead of both histology and culture, allowing for the prompt start of targeted antifungal medication [67]. Additionally, mNGS has shown that it can differentiate between colonization and actual infection, which enhances diagnostic precision in challenging clinical situations [68]. A case study in which mNGS analysis of BAL fluid allowed for the quick detection of pulmonary mucormycosis in a diabetic patient, resulting in early targeted treatment and positive clinical outcomes, further demonstrates its diagnostic potential [69]. However, high costs, limited availability, difficult bioinformatic interpretation, and difficulties differentiating invasive illness from colonization or contamination continue to impede wider clinical application.

Additionally, assays targeting CotH genes increase specificity, as these genes are conserved among pathogenic Mucorales and mostly absent in other fungi. In an investigation, real-time qPCR revealed elevated CotH3 and GRP78 expression in Rhizopus oryzae-infected non-diabetic mice and infected human macrophages. In non-infected diabetic mice, GRP78 expression increased; however, it decreased following infection. Similarly, greater GRP78 levels were seen in sinus tissue from diabetic patients with mucormycosis; these levels dramatically dropped following treatment. Additionally, the researchers found that hsa-miR-16-5p, hsa-miR-93-3p, and hsa-miR-335-5p were downregulated [4]. Nevertheless, compared to these molecular methods, serological diagnostics are still restricted; commercial serological tests are not accessible, and β-D-glucan (BDG) and galactomannan (GM) assays are not beneficial for this class of fungus [70].

### 6.2. Therapeutic Targets

Enhanced knowledge of Mucorales virulence has made it possible to pinpoint particular therapeutic targets that supplement traditional antifungal treatment. The primary therapies for mucormycosis are still liposomal amphotericin B, posaconazole, and isavuconazole, but overall and fungal-associated mortality rates are still high, underscoring the necessity for further, mechanism-based therapeutics [71,72,73]. The CotH–GRP78 interaction is an interesting target for treatments meant to interfere with host–pathogen binding because it is one of the most significant invasion pathways in Mucorales pathogenesis. In an investigation, immunosuppressed and diabetic ketoacidosis (DKA) mice were guarded by anti-CotH3 antibodies against Mucorales species, such as *Rhizopus arrhizus* var. *delemar*. Increased neutrophil recruitment and improved Fc receptor-mediated opsonophagocytic killing were linked to this protection. Monoclonal antibodies also showed wide antifungal action across Mucorales species and demonstrated synergistic benefits when paired with antifungal therapy [16]. Building on these preclinical findings, a recent study revealed the creation of a humanized anti-CotH antibody (e.g., VX-01) that showed a ten-fold increase in binding affinity while retaining equivalent in vitro and in vivo efficacy to its murine counterpart. In addition to having good safety and manufacturability profiles, it inhibited angioinvasion and increased opsonophagocytic killing to facilitate its protective action [74]. These results offer experimental evidence that neutralization of fungal adhesins can reduce virulence and promote ongoing creation of CotH-targeted approaches.

Iron acquisition is another well-known factor of pathogenicity. Preclinical research shows that in murine models of mucormycosis, iron chelation with deferasirox decreased fungal burden and increased survival [75]. Conversely, deferoxamine is contraindicated due to its xenosiderophoric effects, which promote fungal growth. There is currently little clinical evidence for deferasirox, and case reports and small studies have produced conflicting findings, highlighting the need for cautious assessment [76]. A case report of a transfusion-dependent β-thalassemia patient receiving iron chelation therapy with deferasirox and deferiprone who developed quickly progressing rhino-orbital mucormycosis with substantial local expansion and involvement of the central nervous system highlights this ambiguity even more. The infection did not improve despite liposomal amphotericin B, posaconazole, and repeated surgical debridement and the patient passed away within 12 days [77].

Opportunities for host-directed therapy are further highlighted by recent research. During Mucorales infection, HIF-1α signaling is triggered, which concurrently stimulates host pro-inflammatory reactions and encourages *Rhizopus arrhizus* var. *delemar* invasion of airway epithelial cells. Significant activation of host immunological and cellular stress-response pathways, such as hypoxia-associated signaling, was observed in infected lung tissue in a patient-based transcriptome and proteomic investigation of pulmonary mucormycosis [40]. HIF signaling was also found to be upregulated in a transcriptome analysis of COVID-19-associated pulmonary mucormycosis (CAPM) [39]. HIF-1α is a possible host-targeted therapeutic mechanism since its pharmacological inhibition lowers epithelial invasion and increases survival in mouse lung mucormycosis without lowering fungal burden [78]. The use of adjuvant immunomodulation has also been investigated.

In order to boost innate immune defense in refractory conditions, recombinant interferon-γ and granulocyte-macrophage colony-stimulating factor have been delivered; however, the proof for this has mostly come from case reports and small case series instead of controlled trials. For example, a retrospective cohort review revealed that an overall response rate of 82–92% was linked to the utilization of adjunctive sargramostim (yeast-derived recombinant human granulocyte-macrophage colony-stimulating factor [rhu GM-CSF]) in invasive fungal diseases, like Mucorales infections, indicating a potential immunomodulatory adjunctive effect [79]. Furthermore, a case report of *Rhizopus arrhizus* var. *delemar*-induced rhino-orbital cerebral mucormycosis in a patient with diabetic ketoacidosis documented the disease’s development in spite of antifungal medication and recurrent surgical debridement. After the administration of GM-CSF (sargramostim), the patient exhibited decreased periorbital pain and edema, stabilized central nervous system involvement on MRI, and full recovery of vision and ocular function at long-term follow-up [80]. In a case report, invasive cutaneous and peritoneal mucormycosis caused by *Rhizopus microsporus* was treated effectively with a mixture of antifungal therapy, a PD-1 inhibitor, and interferon-gamma. This highlights the potential benefit of customized immunotherapy in refractory cases of invasive mucormycosis [81]. In a similar vein, traditional treatment was ineffective in treating a different case of invasive cutaneous mucormycosis after a severe burn injury. But once interferon-γ (IFN-γ) was administered, the patient’s condition quickly improved, the infection was resolved, and immune function markers like mHLA-DR expression and T-cell proliferation were restored [82].

## 7. Knowledge Gaps and Future Directions

Even with significant progress in identifying the molecular causes of mucormycosis, our knowledge is still disproportionately limited in comparison to the disease’s clinical severity. When contrasted to model fungi like *Aspergillus fumigatus*, functional genomic tools for Mucorales are far less advanced. This restriction is primarily caused by low transformation rates and homologous recombination efficiency, which impede stable gene disruption and focused genome editing in Mucorales. Despite the continued functionality and widespread use of RNA interference-based techniques, CRISPR-Cas9 applications continue to demonstrate variable and species-dependent performance in Mucorales [83]. While *Rhizopus arrhizus* CotH proteins have set the standard for receptor-mediated endothelial invasion, little is known about the regulatory networks controlling CotH expression, the degree of paralog redundancy, and whether other adhesins or released virulence effectors are present. Non-coding RNAs, including putative long non-coding RNAs (lncRNAs), have been found in Mucorales, according to recent transcriptome datasets; nevertheless, their functional significance is yet unknown. Furthermore, the systematic discovery of virulence determinants is still limited by the lack of genome-wide functional screening platforms (such as mutant libraries) [84]. There is currently no comprehensive genome-wide analysis of virulence factors in clinically significant Mucorales species [85,86].

In-depth mechanistic explanations of host–pathogen interactions are also necessary. Although GRP78 is an endothelial receptor that has been validated, it is unlikely to function alone. Co-receptor identities, tissue-specific receptor landscapes, and intracellular signaling pathways that cause endothelial damage, thrombosis, and cytoskeletal remodeling are still not fully understood [16]. Considering that mucormycosis pathology is defined by angioinvasion, careful research is necessary to determine the exact molecular interface between fungal invasion and host coagulation cascades.

Characterization of innate immune responses is lacking, especially in metabolically altered states like diabetes and ketoacidosis. Thoroughly defining the mechanisms behind compromised neutrophil function, complement activation dynamics, cell wall-mediated immune masking, and macrophage handling of germinating spores is still yet to be completed. Adaptive immunity, such as T-cell polarization, antigen specificity, and immunological memory, is not well understood in mucormycosis, in comparison to invasive aspergillosis. There are still unanswered questions regarding host immunometabolic processes and iron metabolism [87]. The interaction between fungal iron acquisition pathways and host regulators like hepcidin and ferritin demand mechanistic clarification, as well as fungal adaptation to hyperglycemic and acidotic microenvironments. Future advancements will rely on metabolomic profiling, dual host–pathogen transcriptomics, integrated functional genomics, and physiologically relevant models that take metabolic comorbidities into account. To transition from descriptive pathobiology to mechanism-oriented therapeutic approaches, these gaps must be filled [11].

## 8. Conclusions

Mucormycosis is a manifestation of an angioinvasive infection that spreads quickly and is caused by tightly controlled molecular interactions between immunocompromised hosts and Mucorales. Combining data from genetic, cellular, and in vivo investigations delineated invasion programs mediated by CotH adhesins, iron assimilation processes adjusted to host metabolic imbalance, and integrated stress-response networks that maintain hyphal survival within harsh microenvironments. Parallel host abnormalities, neutrophil impairment, decreased macrophage fungicidal action, and disrupted cytokine signaling promote unchecked tissue invasion and thrombosis. The COVID-19 pandemic highlighted how viral inflammation, corticosteroid contact, and metabolic disturbances all work together to increase susceptibility. Crucially, mechanistic dissection has shifted the field away from descriptive pathology toward target-centered tactics, such as interference with host–fungal receptor engagement, regulation of iron availability, and using adjuvant immunotherapeutic techniques. Future advancements will rely on holistic multi-omics, reliable genetic manipulation frameworks, and translational validation in order to connect experimental understanding with clinically useful interventions.

## Figures and Tables

**Table 1:** Fungal virulence factors and their molecular function in mucormycosis.

Virulence Determinant	Gene(s)/Molecular Component	Molecular Function	Host Interaction Mechanism	Clinical Relevance Classification	References
CotH	CotH3, CotH7	Surface proteins specific to Mucorales; work as invasins	Direct attachment to host GRP78 on endothelial cells	Antifungal target; candidate molecular diagnostic marker	[4,24]
Iron permease system	FTR1	Reductive iron absorption is facilitated by iron permease.	Helps absorb ferric iron that is released in acidic or iron-overloaded environments	Metabolic virulence factor; potential antifungal target (iron acquisition pathway); risk amplifier in iron-overload states	[25,26]
Xenosiderophore-mediated iron acquisition	Ferrioxamine transport system	Employs deferoxamine as a xenosiderophore for iron acquisition	Deferoxamine-iron complex transported into fungal cells	Latrogenic risk factor pathway (deferoxamine-mediated xenosiderophore activity); therapeutic caution marker in iron-chelation therapy	[27,28]
Calcineurin signaling pathway	CnaA, CnbR	Calcium/calmodulin-dependent phosphatase modulation hyphal expansion	Regulates morphogenesis and stress adaptation	Antifungal drug target; virulence regulator	[29,30]
Heat shock protein 90 (Hsp90)	Hsp90	Stress response protein stabilization by a molecular chaperone	Promotes the survival of fungi under oxidative and heat stress	Therapeutic target (combination therapy)	[31,32]
Cell wall synthesis machinery	Chitin synthases	Cell wall chitin biosynthesis	Keeps the hyphae’s structural integrity intact	Structural antifungal target	[33]
Oxidative stress response enzymes	Superoxide dismutase (SOD), catalase	Detoxification of reactive oxygen species (ROS)	Safeguards against oxidative death caused by neutrophils	Immune evasion factor	[34]

**Table 2:** Host immune responses in mucormycosis.

Immune Component	Key Molecular Mediators	Fungal Trigger	Cellular Response	Outcome	References
GRP78-driven endothelial invasion process	GRP78 (HSPA5), downstream endocytic machinery	CotH surface proteins	Receptor-faciliated fungal endocytosis and endothelial cell damage	Promotes, angioinvasion, vascular thrombosis, tissue necrosis	[13]
TLR2/TLR4–MyD88–NF-κB signals	TLR2, TLR4, MyD88, NF-κB	Spore and germling cell wall components	NF-κB stimulation with TNF-α and IL-6 release	Causes an early innate inflammatory cytokine reaction.	[41]
Dectin-1–SYK identification process	Dectin-1, SYK kinase	β-glucan	Poor SYK signals and moderate cytokine generation	Decreased inflammatory stimulation in contrast to infections caused by Aspergillus	[42]
Neutrophil oxidative burst process	NADPH oxidase, ROS	Hyphal elements	ROS-driven hyphal injury and extracellular death	Neutropenia markedly predisposes to invasive condition	[17]
Macrophage phagolysosomal response to spores	Phagolysosomal enzymes, TNF-α	Sporangiospores	Phagocytosis and suppression of intracellular germination	Early restriction lowers progression to hyphal invasion	[37]
Iron sequestration	Transferrin, ferritin	FTR1-mediated uptake	Host iron attachment limits extracellular iron accessibility	Acidosis decreases transferrin attachment, increasing fungal growth	[5]
IFN-γ–induced macrophage stimulation (Th1 response)	IFN-γ	Antigen presentation following innate stimulation	Increased macrophage antifungal action and cytokine generation	Protective cellular immunity prevents the spread of fungi	[35]

## Data Availability

No new data were created or analyzed in this study. Data sharing is not applicable to this article.

## References

[1] Prakash H., Chakrabarti A. (2021). Epidemiology of Mucormycosis in India. Microorganisms.

[2] Sahu R.K., Salem-Bekhit M.M., Bhattacharjee B., Almoshari Y., Ikbal A.M.A., Alshamrani M., Bharali A., Salawi A., Widyowati R., Alshammari A. (2021). Mucormycosis in Indian COVID-19 Patients: Insight into Its Patho-Genesis, Clinical Manifestation, and Management Strategies. Antibiotics.

[3] Tilwani K., Patel D., Soni P., Wadhwani S., Dave G. (2024). Projecting phytochemical bacoside A anti-mucorale agent: An in-silico and in-vitro assessment. Heliyon.

[4] Seifi Z., Shokohi T., Shafiee M., Mowla S.J., Niknejad F., Siddig E.E., Ahmed A., Abastabar M., Vahedi Larijani L. (2025). The expression of fungal CotH, human glucose-regulated protein 78 (GRP78), and predicted miRNAs in macrophages and diabetic mice infected with *Rhizopus oryzae*. Microbiol. Spectr..

[5] Stanford F.A., Voigt K. (2020). Iron Assimilation during Emerging Infections Caused by Opportunistic Fungi with emphasis on Mucorales and the Development of Antifungal Resistance. Genes.

[6] Monika P., Chandraprabha M.N. (2022). Risks of mucormycosis in the current COVID-19 pandemic: A clinical challenge in both immunocompromised and immunocompetent patients. Mol. Biol. Rep..

[7] García-Carnero L.C., Mora-Montes H.M. (2022). Mucormycosis and COVID-19-Associated Mucormycosis: Insights of a Deadly but Neglected Mycosis. J. Fungi.

[8] Gryganskyi A.P., Golan J., Muszewska A., Idnurm A., Dolatabadi S., Mondo S.J., Kutovenko V.B., Kutovenko V.O., Gajdeczka M.T., Anishchenko I.M. (2023). Sequencing the Genomes of the First Terrestrial Fungal Lineages: What Have We Learned?. Microorganisms.

[9] Itabangi H., Sephton-Clark P.C.S., Tamayo D.P., Zhou X., Starling G.P., Mahamoud Z., Insua I., Probert M., Correia J., Moynihan P.J. (2022). A bacterial endosymbiont of the fungus *Rhizopus microsporus* drives phagocyte evasion and opportunistic virulence. Curr. Biol..

[10] Yin L., Li H., Xing R., Li R., Gao K., Li G., Liu S. (2025). Fungal and Microalgal Chitin: Structural Differences, Functional Properties, and Biomedical Applications. Polymers.

[11] Alqarihi A., Kontoyiannis D.P., Ibrahim A.S. (2023). Mucormycosis in 2023: An update on pathogenesis and management. Front. Cell. Infect. Microbiol..

[12] Tahiri G., Lax C., Cánovas-Márquez J.T., Carrillo-Marín P., Sanchis M., Navarro E., Garre V., Nicolás F.E. (2023). *Mucorales* and Mucormycosis: Recent Insights and Future Prospects. J. Fungi.

[13] Alqarihi A., Gebremariam T., Gu Y., Swidergall M., Alkhazraji S., Soliman S.S.M., Bruno V.M., Edwards J.E., Filler S.G., Uppuluri P. (2020). GRP78 and Integrins Play Different Roles in Host Cell Invasion during Mucormycosis. mBio.

[14] Soliman S.S.M., Baldin C., Gu Y., Singh S., Gebremariam T., Swidergall M., Alqarihi A., Youssef E.G., Alkhazraji S., Pikoulas A. (2021). Mucoricin is a ricin-like toxin that is critical for the pathogenesis of mucormycosis. Nat. Microbiol..

[15] Baldin C., Ibrahim A.S. (2017). Molecular mechanisms of mucormycosis-The bitter and the sweet. PLoS Pathog..

[16] Gebremariam T., Alkhazraji S., Soliman S.S.M., Gu Y., Jeon H.H., Zhang L., French S.W., Stevens D.A., Edwards J.E., Filler S.G. (2019). Anti-CotH3 antibodies protect mice from mucormycosis by prevention of invasion and augmenting opsonophagocytosis. Sci. Adv..

[17] Danion F., Coste A., Le Hyaric C., Melenotte C., Lamoth F., Calandra T., Garcia-Hermoso D., Aimanianda V., Lanternier F., Lortholary O. (2023). What Is New in Pulmonary Mucormycosis?. J. Fungi.

[18] Andrianaki A.M., Kyrmizi I., Thanopoulou K., Baldin C., Drakos E., Soliman S.S.M., Shetty A.C., McCracken C., Akoumianaki T., Stylianou K. (2018). Iron restriction inside macrophages regulates pulmonary host defense against Rhizopus species. Nat. Commun..

[19] Ibrahim A.S., Gebremariam T., Lin L., Luo G., Husseiny M.I., Skory C.D., Fu Y., French S.W., Edwards J.E., Spellberg B. (2010). The high affinity iron permease is a key virulence factor required for Rhizopus oryzae pathogenesis. Mol. Microbiol..

[20] Stanford F.A., Matthies N., Cseresnyés Z., Figge M.T., Hassan M.I.A., Voigt K. (2021). Expression Patterns in Reductive Iron Assimilation and Functional Consequences during Phagocytosis of *Lichtheimia corymbifera*, an Emerging Cause of Mucormycosis. J. Fungi.

[21] Parashar A., Singh C. (2024). Angioinvasive mucormycosis in burn intensive care units: A case report and review of literature. World J. Crit. Care Med..

[22] Gong F., Zheng X., Zhao S., Liu H., Chen E., Xie R., Li R., Chen Y. (2025). Disseminated intravascular coagulation: Cause, molecular mechanism, diagnosis, and therapy. MedComm.

[23] Matiku S.B., Murenzi G., Shaban I., Msonge A.M., Kamafa A.E., Kitua D.W., Kimambo A., Mwakigonja A.R., Massawe E.R. (2024). Mucormycosis: A rare forgotten but fatal disease—A case report and literature review. J. Rare Dis..

[24] Prakash H., Skiada A., Paul R.A., Chakrabarti A., Rudramurthy S.M. (2021). Connecting the Dots: Interplay of Pathogenic Mechanisms between COVID-19 Disease and Mucormycosis. J. Fungi.

[25] Takemura K., Kolasinski V., Del Poeta M., de Sa N.F.V., Garg A., Ojima I., Del Poeta M., de Sa N.P. (2025). Iron acquisition strategies in pathogenic fungi. mBio.

[26] Luo Q., He X., Xu J., Li L., Zhao L., Mu X. (2025). Reduced serum iron levels predict poor prognosis in pulmonary mucormycosis patients: A prospective, case–control study. Sci. Rep..

[27] Dogra S., Arora A., Aggarwal A., Passi G., Sharma A., Singh G., Barnwal R.P. (2022). Mucormycosis Amid COVID-19 Crisis: Pathogenesis, Diagnosis, and Novel Treatment Strategies to Combat the Spread. Front. Microbiol..

[28] Çiçek Y., Dumlu R., Koçak M., Pirdal B.Z., Çelik M., Saltoğlu N., Tekin R., Kömür S., Büyüktuna S.A., Batırel A. (2026). Epidemiological profile and clinical outcomes of patients with mucormycosis: The multicenter retromucor study from Türkiye (2004–2024). Eur. J. Clin. Microbiol. Infect. Dis. Off. Publ. Eur. Soc. Clin. Microbiol..

[29] Park H.S., Lee S.C., Cardenas M.E., Heitman J. (2019). Calcium-Calmodulin-Calcineurin Signaling: A Globally Conserved Virulence Cascade in Eukaryotic Microbial Pathogens. Cell Host Microbe.

[30] Lemhamdi O., Willems E., Baron F., De Prijck B., Caers J., Bouquegneau A., Guiot J., Cousin F., Jadoul A., Rogister F. (2026). Novel diagnostic approaches and therapeutic management of mucormycosis: Insights from a retrospective monocentric cohort study. Front. Med..

[31] Neves-da-Rocha J., Santos-Saboya M.J., Lopes M.E.R., Rossi A., Martinez-Rossi N.M. (2023). Insights and Perspectives on the Role of Proteostasis and Heat Shock Proteins in Fungal Infections. Microorganisms.

[32] Robbins N., Cowen L.E. (2023). Roles of Hsp90 in Candida albicans morphogenesis and virulence. Curr. Opin. Microbiol..

[33] Cheng Q., Dickwella Widanage M.C., Yarava J.R., Ankur A., Latgé J.P., Wang P., Wang T. (2024). Molecular architecture of chitin and chitosan-dominated cell walls in zygomycetous fungal pathogens by solid-state NMR. Nat. Commun..

[34] Santos A.R.D., Fraga-Silva T.F., Almeida-Donanzam D.F., Finatto A.C., Marchetti C., Andrade M.I., Arruda O.S., Arruda M.S.P., Venturini J. (2022). Is the production of reactive oxygen and nitrogen species by macrophages associated with better infectious control in female mice with experimentally disseminated and pulmonary mucormycosis?. PLoS ONE.

[35] Ghuman H., Voelz K. (2017). Innate and Adaptive Immunity to Mucorales. J. Fungi.

[36] Montaño D.E., Voigt K. (2020). Host Immune Defense upon Fungal Infections with Mucorales: Pathogen-Immune Cell Interactions as Drivers of Inflammatory Responses. J. Fungi.

[37] Aberdein J.D., Cole J., Bewley M.A., Marriott H.M., Dockrell D.H. (2013). Alveolar macrophages in pulmonary host defence the unrecognized role of apoptosis as a mechanism of intracellular bacterial killing. Clin. Exp. Immunol..

[38] Shete A., Deshpande S., Sawant J., Warthe N., Thakar M., Madkaikar M., Pradhan V., Rao P., Rohatgi S., Mukherjee A. (2023). Higher proinflammatory responses possibly contributing to suppressed cytotoxicity in patients with COVID-19 associated mucormycosis. Immunobiology.

[39] Dhaliwal M., Muthu V., Sharma A., Raj K., Rudramurthy S.M., Agarwal R., Kaur H., Rawat A., Singh S., Chakrabarti A. (2024). Immune and metabolic perturbations in COVID-19-associated pulmonary mucormycosis: A transcriptome analysis of innate immune cells. Mycoses.

[40] Zhong J., Cai X., Cai Y., Zhao T., Hu D., Sun C., Ni Y., Gu Y., Su X. (2026). Transcriptomic and proteomic profiling of pulmonary mucormycosis reveals a failed activation of host immune response. Front. Immunol..

[41] Montaño D.E., Hartung S., Wich M., Ali R., Jungnickel B., von Lilienfeld-Toal M., Voigt K. (2022). The TLR-NF-kB axis contributes to the monocytic inflammatory response against a virulent strain of *Lichtheimia corymbifera*, a causative agent of invasive mucormycosis. Front. Immunol..

[42] Anaya E.U., Amin A.E., Wester M.J., Danielson M.E., Michel K.S., Neumann A.K. (2023). Dectin-1 multimerization and signaling depends on fungal β-glucan structure and exposure. Biophys. J..

[43] Dos Santos A.R., Fraga-Silva T.F., de Fátima Almeida-Donanzam D., Dos Santos R.F., Finato A.C., Soares C.T., Lara V.S., Almeida N.L.M., Andrade M.I., de Arruda O.S. (2022). IFN-γ Mediated Signaling Improves Fungal Clearance in Experimental Pulmonary Mucormycosis. Mycopathologia.

[44] Loh J.T., Lam K.P. (2023). Fungal infections: Immune defense, immunotherapies and vaccines. Adv. Drug Deliv. Rev..

[45] Soleimanifar N., Assadiasl S., Rostamian A., Abdollahi A., Salehi M., Abdolmaleki M., Barzegari S., Sobati A., Sadr M., Mohebbi B. (2023). Percentage of Th1 and Th17 cells and serum level of IL-17 and IFN-γ cytokines in COVID-19-associated mucormycosis. Med. Mycol..

[46] Das S., Rai G., Gupta C., Gupta N., Arora V., Singh P.K., Mohapatra A., Ansari M.A., Hakami Z.H., Dar S.A. (2024). Mucormycosis in diabetes: A suggested role of Th17 and T regulatory pathways. Infect. Dis. Clin. Pract..

[47] Dandu H., Kumar M., Malhotra H.S., Kumar N., Kumar N., Gupta P., Puri B., Yadav G. (2023). T-cell dysfunction as a potential contributing factor in post-COVID-19 mucormycosis. Mycoses.

[48] Panda S., Sahu M.C., Turuk J., Pati S. (2024). Mucormycosis: A Rare disease to Notifiable Disease. Braz. J. Microbiol..

[49] Lecointe K., Cornu M., Leroy J., Coulon P., Sendid B. (2019). Polysaccharides Cell Wall Architecture of Mucorales. Front. Microbiol..

[50] Pérez-Arques C., Navarro-Mendoza M.I., Murcia L., Lax C., Martínez-García P., Heitman J., Nicolás F.E., Garre V. (2019). *Mucor circinelloides* Thrives inside the Phagosome through an Atf-Mediated Germination Pathway. mBio.

[51] Singh P., Paul S., Shivaprakash M.R., Chakrabarti A., Ghosh A.K. (2016). Stress response in medically important Mucorales. Mycoses.

[52] Morales-Franco B., Nava-Villalba M., Medina-Guerrero E.O., Sánchez-Nuño Y.A., Davila-Villa P., Anaya-Ambriz E.J., Charles-Niño C.L. (2021). Host-pathogen molecular factors contribute to the pathogenesis of Rhizopus spp. in diabetes mellitus. Curr. Trop. Med. Rep..

[53] Radotra B., Challa S. (2022). Pathogenesis and Pathology of COVID-Associated Mucormycosis: What Is New and Why. Curr. Fungal Infect. Rep..

[54] Ibrahim A.S. (2011). Host cell invasion in mucormycosis: Role of iron. Curr. Opin. Microbiol..

[55] Binder U., Maurer E., Lass-Flörl C. (2014). Mucormycosis--from the pathogens to the disease. Clin. Microbiol. Infect. Off. Publ. Eur. Soc. Clin. Microbiol. Infect. Dis..

[56] Gebremariam T., Lin L., Liu M., Kontoyiannis D.P., French S., Edwards J.E., Filler S.G., Ibrahim A.S. (2016). Bicarbonate correction of ketoacidosis alters host-pathogen interactions and alleviates mucormycosis. J. Clin. Investig..

[57] Gebremariam T., Liu M., Luo G., Bruno V., Phan Q.T., Waring A.J., Edwards J.E., Filler S.G., Yeaman M.R., Ibrahim A.S. (2014). CotH3 mediates fungal invasion of host cells during mucormycosis. J. Clin. Investig..

[58] Safiia J., Díaz M.A., Alshaker H., Atallah C.J., Sakr P., Moshovitis D.G., Nawlo A., Franceschi A.E., Liakos A., Koo S. (2024). Recent Advances in Diagnostic Approaches for Mucormycosis. J. Fungi.

[59] Soliman S.S.M., El-Labbad E.M., Abu-Qiyas A., Fayed B., Hamoda A.M., Al-Rawi A.M., Dakalbab S., El-Shorbagi A.A., Hamad M., Ibrahim A.S. (2022). Novel Secreted Peptides From *Rhizopus arrhizus* var. *delemar* With Immunomodulatory Effects That Enhance Fungal Pathogenesis. Front. Microbiol..

[60] Scherer E., Iriart X., Bellanger A.P., Dupont D., Guitard J., Gabriel F., Cassaing S., Charpentier E., Guenounou S., Cornet M. (2018). Quantitative PCR (qPCR) Detection of Mucorales DNA in Bronchoalveolar Lavage Fluid To Diagnose Pulmonary Mucormycosis. J. Clin. Microbiol..

[61] Millon L., Caillot D., Berceanu A., Bretagne S., Lanternier F., Morio F., Letscher-Bru V., Dalle F., Denis B., Alanio A. (2022). Evaluation of Serum Mucorales Polymerase Chain Reaction (PCR) for the Diagnosis of Mucormycoses: The MODIMUCOR Prospective Trial. Clin. Infect. Dis. Off. Publ. Infect. Dis. Soc. Am..

[62] Tang H.M., Chen S.C.-A., Basile K., Halliday C.L. (2025). Development and Evaluation of a Pan-Mucorales Real-Time PCR and a Multiplex Real-Time PCR for Detection and Identification of *Rhizopus arrhizus*, *Rhizopus microsporus*, and *Mucor* spp. in Clinical Specimens. J. Clin. Microbiol..

[63] Brown L., Tschiderer L., Alanio A., Barnes R.A., Chen S.C., Cogliati M., Cruciani M., Donnelly J.P., Hagen F., Halliday C. (2025). The diagnosis of mucormycosis by PCR in patients at risk: A systematic review and meta-analysis. EClinicalMedicine.

[64] Sedik S., Aerts R., Lagrou K., Vanbiervliet Y., Maertens J.A., White P.L., Posso R.B., Schelenz S., Abdolrasouli A., Buil J.B. (2025). Mucorales PCR Testing in Respiratory and Biopsy Samples From Immunocompromised Patients With Invasive Pulmonary Aspergillosis and Other Mold Infections: Results From a Multicenter ECMM Study. Open Forum Infect. Dis..

[65] Rocchi S., Scherer E., White P.L., Guitton A., Alanio A., Botterel F., Bougnoux M.E., Buitrago M.J., Cogliati M., Cornu M. (2025). Interlaboratory assays from the fungal PCR Initiative and the Modimucor Study Group to improve qPCR detection of Mucorales DNA in serum: One more step toward standardization. J. Clin. Microbiol..

[66] Yang F., Zhang Y., Qi B., Chen L., Lin F., Wu J., Gong S., Cao L., Zeng M., Cheng Q. (2025). Clinical Manifestations and Prognosis of Patients With Mucormycosis in Intensive Care Units in Western China: A Multi-Center Retrospective Study. Mycoses.

[67] Wu Y., Yu X., Qi J., Chen Y., Wang R., Liu J., Zhang Y., Zhang W. (2026). Metagenomic next-generation sequencing enables early detection and outcome improvement in perioperative mucormycosis after liver transplantation: A single-center experience. Int. J. Infect. Dis. IJID Off. Publ. Int. Soc. Infect. Dis..

[68] Zhou X., Yang C., Liu X., Wang J., Li Y., Pan L., Peng S., Yu H., Deng X. (2025). Clinical performance of metagenomic next-generation sequencing for distinction and diagnosis of Mucorales infection and colonization. Front. Cell. Infect. Microbiol..

[69] Cheng X., Li T., Wu F., Liu D. (2024). Clinical Manifestation, mNGS Based Diagnosis and Treatment of Pulmonary Mucormycosis with *Rhizopus delemar* in a Diabetic Patient. Infect. Drug Resist..

[70] Montesinos I., Rodriguez-Villalobos H. (2026). Biomarkers in Invasive Pulmonary Fungal Infections: Where Do We Stand?. J. Fungi.

[71] Qin J., Bi H., Tang G., Liu X., Qu J., Lv X., Liu Y. (2025). Real-World Effectiveness and Safety of Isavuconazole Versus Amphotericin B for Patients with Invasive Mucormycosis. Microorganisms.

[72] Engelhard M., Wingen-Heimann S.M., Grüner B., Vehreschild M.J.G.T., Kessel J., Ehrlich S., Spiekermann K., Schalk E., Baermann B.N., Vehreschild J.J. (2026). Clinical and health economic impact of isavuconazole for treatment of invasive aspergillosis and mucormycosis: A retrospective, matched multicentre cohort study in Germany. Infection.

[73] Wang Q., Huang Y., Ma H., Fan G.K. (2024). A case report: Comorbidity of Rhinocerebral mucormycosis and pulmonary aspergillosis with challenging diagnosis. Front. Med..

[74] Gu Y., Singh S., Alqarihi A., Alkhazraji S., Gebremariam T., Youssef E.G., Liu H., Fan X., Jiang W.R., Andes D. (2025). A humanized antibody against mucormycosis targets angioinvasion and augments the host immune response. Sci. Transl. Med..

[75] Ibrahim A.S., Gebermariam T., Fu Y., Lin L., Husseiny M.I., French S.W., Schwartz J., Skory C.D., Edwards J.E., Spellberg B.J. (2007). The iron chelator deferasirox protects mice from mucormycosis through iron starvation. J. Clin. Investig..

[76] Chavan S.S., Mehere Y.D., Khond A.D., Radhakrishnan R. (2021). Role of Deferasirox as an Adjuvant to Parenteral Antifungal Therapy in Invasive Fungal Sinusitis. Egypt. J. Ear Nose Throat Allied Sci..

[77] Jayaraman D., Mahalingam H., Mangam N.G.R., Narasimhan S., Ramanan P.V., Sudhakar K.S., Prasanna Kumar S., Keerthana B., Harikumar M.V., Kindo A.J. (2024). Mucor thriving on iron in beta thalassemia major: A case of rhino-orbital mucormycosis. Pediatr. Hematol. Oncol. J..

[78] Kavaliauskas P., Gu Y., Hasin N., Graf K.T., Alqarihi A., Shetty A.C., McCracken C., Walsh T.J., Ibrahim A.S., Bruno V.M. (2024). Multiple roles for hypoxia inducible factor 1-alpha in airway epithelial cells during mucormycosis. Nat. Commun..

[79] Chen T.K., Batra J.S., Michalik D.E., Casillas J., Patel R., Ruiz M.E., Hara H., Patel B., Kadapakkam M., Ch’Ng J. (2022). Recombinant Human Granulocyte-Macrophage Colony-Stimulating Factor (rhu GM-CSF) as Adjuvant Therapy for Invasive Fungal Diseases. Open Forum Infect. Dis..

[80] Thayanantham P., Kalin-Hajdu E., Dufresne S., Dufresne P.J., Viau-Lapointe J., Tremblay J.A. (2025). Immune-modulating therapy with granulocyte-macrophage colony-stimulating factor (GM-CSF) in refractory rhino-orbital-cerebral mucormycosis—A case report. Front. Immunol..

[81] Mhenni R., Dellière S., Maaouia C.B., Hamane S., Deniau B., Mahévas T., Chaussard M., Coutrot M., Guillemet L., Cupaciu A. (2025). Combined antifungal therapy with immunostimulation for refractory cutaneous and peritoneal mucormycosis caused by Rhizopus microsporus. Diagn. Microbiol. Infect. Dis..

[82] Tawfik D.M., Dereux C., Tremblay J.A., Boibieux A., Braye F., Cazauran J.B., Rabodonirina M., Cerrato E., Guichard A., Venet F. (2022). Interferon gamma as an immune modulating adjunct therapy for invasive mucormycosis after severe burn—A case report. Front. Immunol..

[83] Lax C., Nicolás F.E., Navarro E., Garre V. (2024). Molecular mechanisms that govern infection and antifungal resistance in Mucorales. Microbiol. Mol. Biol. Rev. MMBR.

[84] Tahiri G., Hovhannisyan H., Lax C., Navarro E., Gabaldón T., Nicolás F.E., Garre V. (2025). Genome-wide Discovery of lncRNAs in Mucorales Reveals Essential Roles in Development and Fungal Biology. bioRxiv.

[85] Faiyazuddin M., Sophia A., Ashique S., Gholap A.D., Gowri S., Mohanto S., Karthikeyan C., Nag S., Hussain A., Akhtar M.S. (2023). Virulence traits and novel drug delivery strategies for mucormycosis post-COVID-19: A comprehensive review. Front. Immunol..

[86] Chibucos M.C., Soliman S., Gebremariam T., Lee H., Daugherty S., Orvis J., Shetty A.C., Crabtree J., Hazen T.H., Etienne K.A. (2016). An integrated genomic and transcriptomic survey of mucormycosis-causing fungi. Nat. Commun..

[87] Liang M., Xu J., Luo Y., Qu J. (2024). Epidemiology, pathogenesis, clinical characteristics, and treatment of mucormycosis: A review. Ann. Med..

